# Current views on meningeal lymphatics and immunity in aging and Alzheimer’s disease

**DOI:** 10.1186/s13024-023-00645-0

**Published:** 2023-08-14

**Authors:** Shanon Rego, Guadalupe Sanchez, Sandro Da Mesquita

**Affiliations:** 1 Department of Neuroscience, Mayo Clinic, Jacksonville, FL 32224 USA; 2 Post-baccalaureate Research Education Program, Mayo Clinic Graduate School of Biomedical Sciences, Mayo Clinic, Jacksonville, FL 32224 USA; 3 Neuroscience Ph.D. Program, Mayo Clinic Graduate School of Biomedical Sciences, Mayo Clinic, Jacksonville, FL 32224 USA

**Keywords:** Central nervous system, Meninges, Lymphatic vessels, Innate immune cells, Adaptive immune cells, Aging, Neurodegeneration, Alzheimer’s disease

## Abstract

Alzheimer’s disease (AD) is an aging-related form of dementia associated with the accumulation of pathological aggregates of amyloid beta and neurofibrillary tangles in the brain. These phenomena are accompanied by exacerbated inflammation and marked neuronal loss, which altogether contribute to accelerated cognitive decline. The multifactorial nature of AD, allied to our still limited knowledge of its etiology and pathophysiology, have lessened our capacity to develop effective treatments for AD patients. Over the last few decades, genome wide association studies and biomarker development, alongside mechanistic experiments involving animal models, have identified different immune components that play key roles in the modulation of brain pathology in AD, affecting its progression and severity. As we will relay in this review, much of the recent efforts have been directed to better understanding the role of brain innate immunity, and particularly of microglia. However, and despite the lack of diversity within brain resident immune cells, the brain border tissues, especially the meninges, harbour a considerable number of different types and subtypes of adaptive and innate immune cells. Alongside microglia, which have taken the centre stage as important players in AD research, there is new and exciting evidence pointing to adaptive immune cells, namely T and B cells found in the brain and its meninges, as important modulators of neuroinflammation and neuronal (dys)function in AD. Importantly, a genuine and functional lymphatic vascular network is present around the brain in the outermost meningeal layer, the dura. The meningeal lymphatics are directly connected to the peripheral lymphatic system in different mammalian species, including humans, and play a crucial role in preserving a “healthy” immune surveillance of the CNS, by shaping immune responses, not only locally at the meninges, but also at the level of the brain tissue. In this review, we will provide a comprehensive view on our current knowledge about the meningeal lymphatic vasculature, emphasizing its described roles in modulating CNS fluid and macromolecule drainage, meningeal and brain immunity, as well as glial and neuronal function in aging and in AD.

## Background

The meningeal tissue of the central nervous system (CNS) harbours a crucial yet overlooked network of lymphatic vessels. In 2015, two initial and ground-breaking papers, led by independent groups, carried out the first in-depth structural and functional studies of the meningeal lymphatic vasculature [1, 2], confirming some of the initial elementary, yet unfortunately discredited observations made by Paolo Mascagni in the 18^th^ century [3]. This (re)discovery has triggered a cascade of scientific curiosity and generalized efforts to elucidate the changes and workings involving this lymphatic system that continuously drains the CNS. The growing awareness about a meningeal lymphatic vascular network that establishes a direct and functional connection between the CNS meninges and the peripheral lymphatic system, also contributed to the ongoing challenge of the traditional CNS immune privilege concept [4, 5]. Herein, we will be delving on some of the recent studies that have widened our understanding about the roles of the meningeal lymphatics, namely in modulating the intricate, constant, and fine-tuned neuroimmune crosstalk at the brain borders and parenchyma, either in steady state or in disease. In the next sections of this review manuscript, we will provide an update on the structural and functional features of the rodent and primate meningeal lymphatic vascular network, the nature of the neuroimmune crosstalk between meningeal immune and brain cells, and the changes in meningeal lymphatic function and immunity in aging. We will then discuss recent studies that provide new insights on the immune contributions to Alzheimer’s disease (AD) pathophysiology and, finally, provide an overview of the body of scientific evidence that strengthens the link between altered meningeal lymphatic drainage, dysregulated and/or deleterious neuroimmune responses, neuropathology, and neurodegeneration in the context of AD.

## The meningeal lymphatic vascular system

**Fig. 1:**
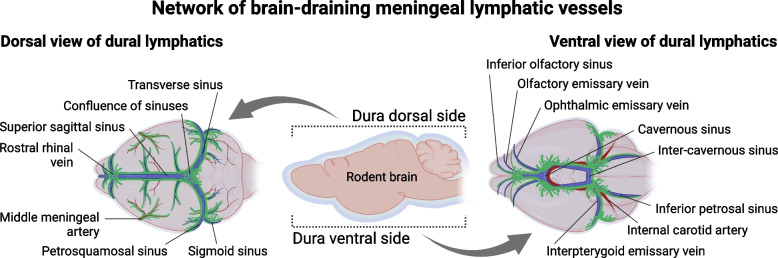
Scheme showing the lymphatic vessel networks surrounding blood vessels in the dorsal (illustration to the left) and ventral (illustration to the right) meningeal dura

In mammalian organisms, the meninges wrap around the entire CNS tissue, encompassing the brain and the spinal cord, and are composed of three distinct layers: the innermost layer, known as the pia mater, the arachnoid (mostly acellular and linked to the pia via trabeculae), and the outermost layer, the dura mater, which is in direct contact with the skull [6–8]. Recently published data suggests the existence of a fourth layer that divides the subarachnoid space into two compartments, named the subarachnoid lymphatic-like membrane; yet, more studies need to be performed to fully discern whether this represents a fully distinct meningeal layer [7]. In healthy conditions, the fully matured lymphatic vasculature of the CNS meninges is spatially limited to the dura mater and does not physically interact with the brain tissue [1, 9, 10] (Fig. 1). Lymphatic vessels are mostly found along the dura mater’s large calibre venous and arterial blood vessel structures and are formed by *bona fide* lymphatic endothelial cells (LECs) that express all the hallmark proteins, including prospero homeobox 1 (PROX1), vascular endothelial growth factor receptor 3 (VEGFR3), lymphatic vessel endothelial hyaluronan receptor-1 (LYVE-1), podoplanin, and C–C motif chemokine ligand 21 (CCL21) [1]. Meningeal lymphatic vessels have been identified in zebrafish, rodents, non-human primates, and humans, although much remains unknown about their development, complexity and function in each organism [1, 10–13]. In the periphery, the primordial LECs appear during embryonic stages. Initial studies involving rodent models have shown that embryonic venous precursor cells, from the cardinal veins, start expressing *Prox1* at embryonic gestation day 9.5 and give rise to a population of LECs that, in return, assemble to produce the lymph sacs and then the lymphatic vessel networks in various peripheral tissues [14, 15]. Similar processes of embryonic development of peripheral lymphatic vasculature are conserved between zebrafish, rodents and primates (reviewed in [16]). However, experiments involving rodents have shown that, in contrast to most peripheral tissue lymphatics, the intracranial and spinal meningeal lymphatic vascular system develops postnatally [17]. LECs and sprouting lymphangiogenesis are observed at the base of the skull within the first 8 days after birth, first in the vicinity of the pterygopalatine and middle meningeal arteries, and later in the sigmoid sinus. The entire murine meningeal lymphatic network is fully developed and functional around postnatal day 28, through a process that is dependent on vascular endothelial growth factor C (VEGF-C) signalling through its cognate receptor VEGFR3 expressed on LECs [17]. Whether a similar meningeal lymphatic developmental process is observed in non-human primates and humans is a subject that warrants further investigation.

Several groups have now demonstrated that the meningeal lymphatic vessels play an important role in cerebrospinal fluid (CSF) drainage into the peripheral lymphatic system, via the cervical lymph nodes (LNs), not only in rodents, but also in humans [1, 10, 18–21] (Fig. 1). This was possible through newly developed magnetic resonance imaging (MRI) protocols that can successfully distinguish lymphatic from blood flow at the meninges, as well as drainage of CSF content into the cervical LNs of primates and humans [10, 12, 20]. These MRI modalities, alongside in vivo and ex vivo fluorescence imaging techniques involving rodent models [18, 21, 22], have elegantly shown that the meningeal lymphatic outflow process happens continuously and at a considerable rate [18, 21]. Recent studies involving mice are also pointing to a circadian rhythmicity of meningeal lymphatic drainage into the superficial cervical LNs, which seems to happen at a higher rate during the animal’s active period [23]. However, more work needs to be performed in this area of research to fully understand the molecular underpinnings of meningeal lymphatic drainage along the sleep-wake cycle. Interestingly, recent studies have revealed a functional crosstalk between the meningeal lymphatic and glymphatic systems, which are anatomically segregated, but connected through their roles of mediating fluid drainage from the brain and spinal cord [9, 18, 23]. The glymphatic system is comprised of the brain perivascular routes formed by the glia limitans and the blood endothelial basement membrane and has been put forward as one of the possible mechanisms responsible for molecular exchange between the CSF and the brain interstitium [24–26]. Despite remaining to some degree controversial [27, 28], one of the currently accepted working models states that glymphatics cleanse the brain parenchyma by “washing” it with CSF that enters through periarterial spaces, diffuses, interacts with the interstitial fluid (ISF) and then flows out of the parenchyma and into the subarachnoid space via perivenous spaces [24, 28]. The ISF containing “wasteful” CNS molecular content is then able to mix with the subarachnoid CSF and later reach the meningeal dura mater to be drained by the meningeal lymphatic vessels into the cervical LNs. Although there is some debate about the physical routes of communication between the subarachnoid space and the meningeal dura, the fact is that a process of molecular exchange between these two compartments takes place, most likely via thinner and more permeable regions of the meningeal arachnoid layers [7, 10, 29]. Upon arrival to the vicinity of the dural lymphatics, CSF content is initially picked up by the initial lymphatic vessels (also called capillary lymphatics), which are thin-walled, formed by a monolayer of LECs that express high levels of LYVE-1 and CCL21, and are bound by button-like junctions [1, 17, 21]. The highly permissive initial lymphatics then converge into larger collecting-like lymphatic vessels at the more basolateral regions of the meningeal dura (Fig. 1). Collecting lymphatics are formed by LECs (expressing lower levels of LYVE-1) bound by zipper-like junctions, contain functional valves that prevent the backflow of drained lymph content, but only become ensheathed by smooth muscle cells as they exit the skull [2, 17, 21]. A more comprehensive mapping of the rodent and human meningeal lymphatic network has revealed that, alongside the network of initial and collecting-like lymphatics adjacent to the transverse, sigmoid, and petrosquamous sinuses, both species also present a complex lymphatic network at the midanterior skull base, along the dural cavernous sinus (Fig. 1). In the adult rodent, all the different bundles of meningeal collecting lymphatic vessels descend along the major meningeal arteries and veins to exit the skull through the basal foramina to connect to the peripheral lymphatic system [10, 17, 30]. Collectively, we now have evidence pointing to two major routes of CNS content drainage into the peripheral lymphatic system in physiological conditions: one that is apparently not mediated by meningeal lymphatics, where fluid and molecules exit the skull through the cribriform plate along cranial nerves to meet the extra-cranial nasal lymphatics that drain into the superficial cervical LNs [10, 31], and a second, truly mediated by the meningeal lymphatic network, that continuously drains CNS fluids and meningeal immune cells into the deep and superficial cervical LNs [9, 10, 21, 30] (Fig. 1). However, our understanding of the meningeal lymphatic network, and of CNS lymphatic drainage as a whole, might have to be revisited in years to come, as new experimental evidence is generated.

## New insights into meningeal immune composition and responses

In homeostatic conditions, the healthy mammalian brain parenchyma is highly populated by resident innate myeloid immune cells (reviewed in [32, 33]), but harbours only very scarce numbers of adaptive immune cells. Adaptive immune cells residing in the brain parenchyma can be found either at periventricular regions, closely adjacent to blood vessels, or next to the highly vascularized leptomeninges [34–37]. Interestingly, and contrarily to the CNS parenchymal tissue, a vast display of different lymphocyte types and subtypes, as well as different professional innate phagocytes and antigen-presenting cells (APCs), are found in substantial numbers at the CNS border tissues (reviewed in [5]), including the choroid plexus and meninges. In the next paragraphs we will focus on particular populations of resident meningeal innate and adaptive leukocytes and discuss the mechanisms through which they are capable of modulating neural cell responses and brain function.

Immune cells populate the meninges early on during developmental stages arriving either from the blood or the dural-adjacent skull bone marrow (connected to the dura via specialized vascular channels) [38–43]. In the meningeal dura, innate immune cells, including macrophages, dendritic cells (DCs), neutrophils, innate lymphoid cells (ILCs), natural killer (NK) cells, and mast cells, are represented at a higher frequency when compared to adaptive immune cells. Of note, macrophages are the most prevalent innate immune cell type in the dura [22, 39, 44]. Like their “close cousins”, the brain perivascular macrophages (PVMs), and contrarily to brain resident microglia, dural macrophages are thought to originate from yolk sac primitive myeloid cells that express cluster of differentiation 206 (CD206). Interestingly, in mice, the transcriptional signatures of microglia and of meningeal macrophages and brain PVMs (jointly called border-associated macrophages; BAMs) become distinct at embryonic day 12.5, mostly due to unparalleled molecular cues present at the different anatomical locations [38, 45]. Noteworthy, the primordial postnatal meningeal macrophages are the source of brain PVMs through a migration process that is dependent on the expression of adhesion molecules like Talin-1 by myeloid cells, and requires functional arterial vascular smooth muscle cells [46]. Recent lineage tracing and adoptive transfer experiments in adult mice have also shown that skull bone marrow derived myeloid precursors can dive into the dura, using specialized vascular channels [47], and replenish the pool of dural resident macrophages and neutrophils [42]. These observations opened a whole new window of possibilities when it comes to the maintenance and turnover of meningeal macrophages, and of meningeal immune cells overall. The process of skull bone marrow-derived innate immune cell replenishment of the meninges was observed in steady-state conditions, and exacerbated in models of increased neuroinflammation, suggesting that this might represent a previously unappreciated disease-associated mechanism [41, 42]. Future studies should aim at understanding whether this process can be harnessed to promote protective immune responses in the CNS. Broadly, dural macrophages can be separated into two clusters based on their expression of major histocompatibility complex class II (MHC-II). High and low MHC-II expressing macrophages have their own gene signatures indicating that they may have divergent roles in either steady state or disease conditions [39, 45]. Interestingly, the establishment of a balance between BAMs expressing high or low MHC-II levels happens during early postnatal stages and seems to be dependent, at least in part, on myeloid cell-specific gene expression downstream of the *fms*-intronic regulatory element enhancer, including the expression and signalling by colony stimulating factor 1 receptor (CSF1R) [48, 49]. Whether altered CSF1R signalling modulates other aspects of meningeal immunity and vascular responses are subjects that warrant further investigation. In a mouse model of hypertension induced by angiotensin 2, BAMs have been shown to contribute to increased levels of reactive oxygen species at the blood-brain barrier (BBB), and mediate, at least in part, the neuroinflammatory response that culminates in increased arteriole and venule permeability and worsened cognitive dysfunction [50, 51]. Yet, a growing body of evidence also points to meningeal macrophages as essential first responders to pathogens that are trying to access the CNS. Dural macrophages act as immune sentinels that phagocytose invading microbes, namely viruses, and release different cytokines, such as interferon-beta (IFN-β), -gamma (IFN-γ) and tumor necrosis factor (TNF), to recruit peripheral effector immune cells that are essential to resolve the infection [52]. Mice infected with lymphocytic choriomeningitis virus, failed to mount an efficient immune response in the absence of CD163 expressing meningeal macrophages. In this context of viral infection, a reduction in dural MHC-II^high^ macrophages was linked to a protective immune response, whereas an exacerbation of viral load in the brain and higher mortality were observed in mice depleted from meningeal macrophages and T cells [53]. This suggests that antigen presentation by MHC-II^low^ meningeal macrophages (potentially to CD8 T cells) might be crucial for preventing worse outcomes in brain viral infections.

DCs represent another main professional APC type in the meningeal dura that have the capacity to capture CSF antigens, migrate to initial lymphatics in a C–C motif chemokine receptor type 7 (CCR7) dependent manner, and drain to cervical LNs where they present antigens to promote T cell responses during neuroinflammation [21, 54]. By phagocytosing microbes within the CNS and its borders, DCs (alongside macrophages) play an important role in the context infection by being the bridge between the innate and adaptive immune system, and potentiating exacerbated, long-lasting and often fatal pathogen-specific meningeal immune responses [52, 55–57]. Within the meninges, one can find three main subtypes of DCs; conventional DCs type I and II, and plasmacytoid DCs (pDCs) [37, 39]. pDCs can produce high levels of type I IFN cytokines during viral infections, namely IFN-α, and present phagocytosed and processed antigens to the surrounding CD4 and CD8 T cells. Conventional DCs type I and II in the gut and spleen acquire distinct gene signatures and functional properties according to the expression of specific transcription factors, namely basic leucine zipper ATF-like transcription factor 3 and interferon regulatory factor 8 by conventional DCs type I, and interferon regulatory factor 4 by conventional DCs type II. Broadly, conventional DCs type I are capable of cross presenting antigens via MHC-I to CD8 T cells, while conventional DCs type II present antigens via MHC-II to CD4 T cells (reviewed in [58–60]). Little is known, however, about the involvement and potential roles of meningeal dural resident DCs when it comes to the maintenance of CNS immune tolerance and beneficial immune surveillance. In a steady state condition, neutrophils and monocytes originate from the skull bone marrow and infiltrate the meninges. Interestingly, monocyte-derived dendritic cell progenitors were also found in the skull bone marrow, pointing to another origin for dural DCs besides the blood [42]. However, more studies have to be performed in order to fully understand the ontogeny of dural neutrophils, monocytes, and DCs and how they influence immune responses in the meninges and brain in health and in disease.

The meningeal dura harbours other innate immune cells, namely ILCs, NKs, and mast cells [22, 44], whose roles have only recently been systematically explored. Type 2 ILCs represent the most abundant group of ILCs in the dura and, as it is characteristic of this type, have shown to respond to IL-33, express the transcription factor GATA binding protein 3, and to contribute to the local production of IL-4, IL-5 and IL-13 [61–63]. Interestingly, enhancing type 2 ILCs’ responses in models of spinal cord injury and cerebral ischemia was linked to lessened neuroinflammation and better disease outcomes [62, 63]. Recently published studies have also shined some light on the contributions of meningeal NKs, type 1 ILCs, and mast cells to neuroinflammation and the modulation of brain function. Dural NKs and type 1 ILCs contribute to the local steady-state production of IFN-γ and acetylcholine, influencing cognitive behaviour via the modulation of the frequency of GABAergic neurotransmission on hippocampal and cortical pyramidal neurons, and anxious-like behaviour by changing the dopamine levels in the hippocampus via the reduction of dopaminergic neuronal activity in the ventral tegmental area [64]. Mast cells present in the vicinity of meningeal dural blood vessels and have been closely implicated in the modulation of brain function and in migraine development. Notably, recent work shows that early life stress has a profound impact on meningeal inflammation and drives dural mast cell activation, which then promoted anhedonic-like behaviour in mice [65–67]. However, we have a very little understanding about the nature of the systematic interactions between different subpopulations of dural immune cells, including mast cells, and how altered communication between them can predispose to increased proinflammatory cytokine secretion (e.g., TNF and calcitonin gene-related peptide) and mast cell activation in migraine.

There is a constant communication between innate and adaptive immune cells at the CNS meningeal interface. Recent studies have indicated that the density of APCs and T cells is higher in the vicinity of the dural sinuses, which are large fenestrated vascular structures that facilitate the intense interaction between meningeal resident immune cells and blood cells and molecules [39]. In fact, the dural venous sinuses serve as a site of active immune surveillance where APCs can acquire either blood- or brain-borne antigens (brought by the CSF that reaches the dura) and present them locally to T cells [39]. T and B cells make up the vast majority of the resident adaptive immune cells within the mammalian brain meningeal dura [37, 68], and mice lacking a functional adaptive immune system, and by extension missing competent T and B cells in the meninges and draining cervical LNs, exhibit significant impairments in exploratory, social and cognitive behaviours [69–71]. Interestingly, the adoptive transfer of secondary lymphoid organ lymphocytes or T cells alone into mice lacking mature adaptive immune cells, resulted in a rescue of the behaviour deficits, directly implicating lymphocytes in the regulation of brain intrinsic processes [69, 70, 72, 73]. The adult steady-state pool of meningeal T cells is restocked by blood circulating T cells that extravasate via the dural sinuses and acquire a tissue-resident phenotype [39, 40]. Until now, brain/meningeal resident adaptive immune cells, namely conventional CD4 T cells and unconventional T cell receptor gamma/delta (TCRγδ) T cells, have been implicated in the modulation of homeostatic neural processes, including neurogenesis, microglial maturation and activation, synaptic plasticity, and neuronal activity, via the secretion of neuromodulatory molecules, such as interleukin 4 (IL-4), IL-17, and IFN-γ [40, 70, 74–77]. Mucosal-associated invariant T (MAIT) cells, a subpopulation of T cells thought to be only present in the skin and mucosal barriers, are also found in the brain meninges [78] Meningeal MAIT cells express antioxidants, namely selenoprotein P, that contribute to the regulation of the surrounding immune niche by neutralizing reactive oxygen species, and thus preserving meningeal inflammation and barrier integrity. Interestingly, mice deficient for the MHC-I-related gene (or *Mr1*), consequently lacked MAIT cells, and presented with learning and memory deficits [78]. Regulatory T cells (Tregs) overexpressing transcription factor forkhead box P3 and highly responsiveness to IL-2 also populate the steady state meninges and brain [37, 75], and have been closely implicated in modulating the neuroinflammatory responses, alongside conventional effector T cells and unconventional TCRγδ T cells, in mouse models of pain hypersensitivity, traumatic brain injury, ischemic injury and stroke, CNS infection, and autoimmune disease mediated by encephalitogenic T cells [21, 36, 79–83].

The mammalian dura (of rodents and non-human primates alike) harbours a diverse repertoire of B cells, encompassing both progenitor, early and resident mature B cells, as well as plasma cells, that in physiological conditions are mostly supplied by the bone marrow of the skull calvaria via specialized vascular channels [43, 68]. Recent studies have provided a more comprehensive view on the steady state murine meningeal B cell pool by using single-cell RNA sequencing (scRNA-seq). Using this technique, researchers were able to characterize the different B cell maturation stages in the meningeal dura and put forward C–X–C chemokine ligand 12 (CXCL12) signalling via C–X–C chemokine receptor 4 (CXCR4), alongside IL-7 signalling via IL-7 receptor (both receptors expressed by B cells), as the mechanisms involved in their survival, differentiation, and maintenance [43, 68]. Interestingly, data strongly suggests that the meningeal dural interstitium, enriched with brain-derived molecules like myelin components and proteins, provides an additional checkpoint to enforce B cell tolerance, in the event B cells evade previous tolerance checkpoints in the bone marrow. This idea is supported, in part, by the presence of myelin oligodendrocyte glycoprotein (MOG) specific and immunoglobulin M (IgM) producing B cells in the meningeal dura, that greatly increase when the dural MOG levels are experimentally reduced [68]. It will be important to understand whether myelin-specific T cells also undergo a similar negative selection process at the meninges. At this point, the data seems to suggest that, under physiological conditions, the immune cells of the meningeal dural interact to foster CNS immune tolerance, and to functionally counteract the formation of encephalitogenic B cell responses. On the other hand, B cells aid in the fortification of the meningeal barrier against infectious agents via their antibody-producing capacity. Meningeal B cells can differentiate into plasma cells that secrete large amounts of IgA into the perisinusal space, a process that seems to be conserved in rodents and humans [84]. Experimental data, obtained by B cell receptor sequencing, also supported the notion that B cell clones originating from the gut upon recognition of microbes/pathogens migrate to the dura, where they become IgA secreting plasma cells that prevent CNS infection by fungi [84]. On the other hand, in the context of brain injury by stroke, meningeal B cells seem to play a deleterious role. The extended recruitment of B cells that undergo isotype switching and secrete antibodies at the neuropil adjacent to the stroke lesion has been linked to worsened and persistent cognitive impairment [85]. Altogether, these data further bolster an exciting new concept of a gut-neuroimmune axis that shapes meningeal adaptive immunity and continuously modulates neural integrity and function either in homeostatic conditions or disease states, throughout the entire mammalian lifetime.

The meningeal immune cells’ ability to exert their homeostatic function in the CNS and its border tissues also relies on meningeal lymphatic drainage into the cervical LNs. A recent study has shown that mice lacking meningeal lymphatic vessels present reduced levels of IL-12 and calcitonin gene-related peptide, but increased levels of the proinflammatory and the mast cell activator CCL2. This was accompanied by a trend in higher mast cell density in the dura and linked to an aberrant spiking activity of meningeal trigeminal neuronal afferents [65]. Additional work will be needed to fully understand whether and how meningeal lymphatic drainage influences the immune/inflammatory responses that underly migraines. Preventing the lymphatic drainage of CNS/meningeal derived antigens, and trafficking of leukocytes into/out of the cervical LNs, either by surgical ligation of the afferent lymphatics to the deep cervical LNs (or resection of the same LNs), treatment with the sphingosine-1-phosphate receptor modulator FTY720, or deficiency in CCR7 expression, have been linked to altered meningeal T cell responses and the development of cognitive deficits [18, 21, 37, 74, 86]. Interestingly, adult mice deficient for CCR7 in hematopoietic lineage cells presented a modest but significant impairment in two behavioural tests that assess spatial learning and memory, namely the novel location recognition test and the Morris water maze [37]. CCR7 is expressed at a great extent by meningeal conventional T cells, B cells, macrophages, and DCs, but not by microglia, and binds to CCL19 and CCL21 secreted mostly by dural LECs, to promote immune cell egress via lymphatics into the cervical LNs [37, 87]. The cognitive impairment in CCR7^*–/–*^ mice was accompanied by an aberrant accumulation of CD4 and CD8 T cells in the dura and, conversely, a steep decrease in T cell content in the cervical LNs. Interestingly, an increased frequency of Tregs within CD4 T cells was observed in the meninges and cervical LNs of CCR7^*–/–*^ mice, mirroring what is observed in 2-year-old mice and pointing to decreased lymphatic drainage as a previously unappreciated mediator of aging-related immune decay in the CNS [37]. In the next section, we will go over the effects of aging on meningeal lymphatic drainage and immunity and how these changes can seriously impact brain physiology and function.

## Changes in meningeal lymphatics and immunity during aging

**Fig. 2:**
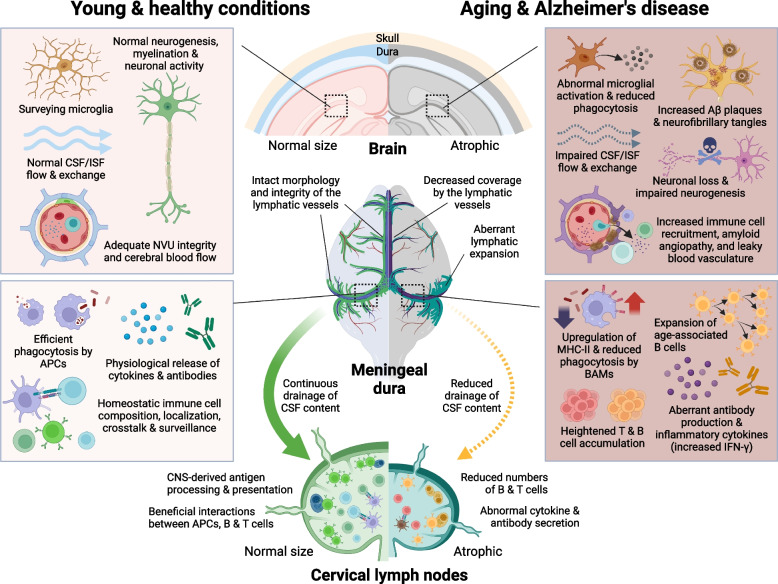
Scheme depicting cellular and molecular processes that happen at the level of the brain parenchyma, meningeal dura, and central nervous system (CNS)-draining cervical lymph nodes in the young and healthy state (illustrations to the left), or during aging and in Alzheimer’s disease (illustrations to the right). Amyloid beta, Aβ; antigen-presenting cells, APCs; border-associated macrophages, BAMs; cerebrospinal fluid, CSF; interferon-gamma, IFN-γ; interstitial fluid, ISF; major histocompatibility complex class II, MHC-II; neurovascular unit, NVU

Aging, by definition, is a combination of processes that drive the progressive deterioration of different organs and systems in the mammalian body over time. Both the immune and the central nervous systems are greatly affected by aging, which represents the main risk factor for different neurological disorders (reviewed in greater detail in [88–91]). It has been recently shown that aging also leads to changes in meningeal lymphatic vascular function that can then contribute to the decay in brain processes by affecting fluid drainage and/or the immune composition and activation in the CNS [18, 21, 30, 37, 92]. In aged mice, the meningeal initial lymphatics exhibit a significant reduction in length and diameter, whereas the downstream collecting-like lymphatics expand and become lymphoedematous, leading to reduced CSF drainage into the cervical LNs [18, 22, 30] (Fig. 2). Interestingly, the decay in meningeal lymphatic function observed in aged mice follows the drastic reduction in fluid exchange through the glymphatic pathway [93]. In fact, experiments involving aged mice have shown an intricate connection between reduced meningeal lymphatic outflow, impaired glymphatic fluid influx/efflux, and cognitive function. Therapeutic delivery of the lymphangiogenic VEGF-C into the CNS of aged mice led to enhanced meningeal lymphatic drainage into the deep cervical LNs, and concomitant improvements in glymphatic influx of CSF into the brain, and in hippocampal-dependent spatial learning and memory [18]. Similarly, MRI data from human patients, including elderly individuals, revealed a connection between meningeal lymphatic morphological and functional impairments, atrophy of the cervical LNs, and reduced glymphatic influx/efflux in older patients [94, 95]. Yet, the exact timeline and mechanisms underlying the deterioration of the meningeal lymphatic system, as well as its connection to dampened glymphatic function in aging, remain an intriguing topic of investigation. Attempting to elucidate this matter, a recent study has linked the lymphatic dysfunction observed in aging to concomitant alterations in meningeal T cell responses [96]. Transcriptomic analysis of the meninges of aged mice showed an upregulation in genes responsible for T cell recruitment and retention in the meninges, such as *Ccl3*, *Ccl5*, *Ccl8*, and *Cxcl12* [39, 97]. Contrarily to what is observed in the young dura, where T cells accumulate along perisinusal areas, the T cells in old mice are scattered across the entire dural stroma, occupying spaces both close to and far from the venous sinuses [39]. Aged mice also present marked cervical LN atrophy, due to loss of T and B cell content, and have significantly more resident CD4 and CD8 T cells in the meninges, which contributes to the increased levels of IFN-γ in this tissue [37, 39] (Fig. 2). Of note, increasing IFN-γ signalling in the dura of adult mice led to poor meningeal lymphatic vessel integrity, leakiness, and reduced drainage into the cervical LNs. On the other hand, prolonged depletion of IFN-γ from the circulation in aged mice was linked to improved meningeal lymphatic drainage into the deep cervical LNs [96]. However, it remains unclear whether the appearance of deficits in lymphatic drainage during aging precede or mediate the increase in dural T cells and in T cell-derived IFN-γ. On this topic, a recent study investigating the changes in meningeal T cell lymphatic egress in aging revealed a reduction in CCR7 expression by T cells found in the aged dura [37]. Meningeal T cells are highly dependent upon the CCR7 signalling pathway, via its ligands CCL19 and CCL21 produced by LECs, for lymphatic egress from the meninges towards the cervical LNs [21, 37]. Interestingly, loss of hematopoietic CCR7 in adult mice was enough to recapitulate the increased numbers of both effector (IFN-γ-producing) and Tregs in the dura that is seen in old mice, as well as the reduction in glymphatic influx of CSF and an early appearance of cognitive deficits [37, 39]. However, the higher numbers of IFN-γ-producing T cells in the meninges of adult CCR7^*–/–*^ mice (alongside the heightened Treg response) were not sufficient to impair meningeal lymphatic morphology or its capacity to drain CSF into the deep cervical LNs [37], suggesting that other aging-related immune changes that are not recapitulated in the CCR7^*–/–*^ mouse model (e.g., altered dural innate immune responses) might be required to potentiate (or intermediate) the deleterious effects of increased IFN-γ on the meningeal lymphatic system. More experiments will be needed to fully understand the functional links between heightened effector T cell and Treg responses in the aged meninges and, holistically, how altered meningeal immunity contributes to defective lymphatic vessel morphology and drainage.

Meningeal B cells also undergo drastic changes with aging. Like T lymphocytes, B cells also accumulate within the meninges of old mice [43], and recent transcriptomic analysis of the entire dura of aged mice acknowledged the upregulation of numerous B cell-associated antibody genes [98]. Interestingly, elegant experiments combining scRNA-seq and B cell receptor sequencing revealed an enrichment in B cells whose gene signature resembled that of blood circulating B cells in the aged dura. This cluster was aptly named age-associated B cells (ABCs) and included cells that showed features of antigen-experienced B cells (with an increased in B cell clonality), overexpressed the apolipoprotein E (*Apoe*), *Cd2*, galectin 1, Ig heavy constant Mu, Ig kappa constant, lymphocyte antigen 6 family member A, spleen associated tyrosine kinase (*Syk*), and zinc finger and BTB domain containing 20 genes [43] (Fig. 2). Likewise, the plasma cells from young and old mice also presented distinct features, with higher production of IgA by young meningeal plasma cells and higher production of IgG and IgM by their aged counterparts. Aged mice dural plasma cells also presented higher clonality and a significant overlap with ABC clones, suggesting that a portion of plasma cells result from ABC terminal differentiation in the aged dura [43]. It would be interesting to investigate whether decreased meningeal lymphatic drainage, alongside potential changes in germinal center B cell responses at the CNS-draining cervical LNs, are somehow involved in the generation of ABCs in elderly mammalians.

Besides T and B cells, aging also causes major changes in meningeal innate immunity. Resident type 2 ILCs increase in numbers in the aged brain meninges but show a decreased capacity to secrete IL-5 and IL-13. Interestingly, aging does not impact on the type 2 ILCs’ responsiveness to IL-33 and enhancing the response by the brain-border associated ILCs, either by administration of IL-33, exogenously activated type 2 ILCs directly into the brain ventricles, or their secreted cytokine IL-5 was shown to have a beneficial impact on the memory function of old mice [61]. A more thorough and unbiased characterization of the CNS immune landscape by mass cytometry also revealed a decreased frequency of pDCs and CD24^+^ conventional DCs type II, that was accompanied by an increased abundancy of CD135^+^ conventional DCs type II [44]. Regarding BAMs, aged mice showed increased frequency of CD38^+^MHC-II^+^CCR2^–^ and CD38^–^MHCII^+^CCR2^–^, and a corresponding decrease in CD38^+^MHC-II^–^CCR2^–^ and CD38^–^MHC-II^+^CCR2^+^, when compared to their younger counterparts [44]. Accordingly, two independent studies employing bulk RNA-seq data of whole meningeal dural preparations, have identified significant gene expression modules and pathways that point to alterations in dendritic cells, macrophages, and monocytes in aging [97, 98]. In the aged leptomeninges and dura, macrophages remain CD206^+^, express lower levels of LYVE-1, become MHC-II^high^, and contribute to higher levels of triggering receptor expressed on myeloid cells 2 (*Trem2)* gene expression [39, 45, 99] (Fig. 2). Despite some controversy about the changes in meningeal BAMs numbers in aged mice, evidence suggests that these remain similar, despite the apparent acquisition of a phenotype indicative of higher proliferation and activation [45, 97, 99]. Interestingly, this characteristic signature of aged meningeal BAMs was closely associated with extracellular matrix remodelling at the basement membrane of brain penetrating blood vessels and impaired CSF glymphatic influx [99]. The therapeutic delivery of CSF1 into the aged mice CNS acted as a rejuvenation factor for leptomeningeal and brain BAMs, reverted the extracellular matrix anomalies and improved glymphatic function [99]. It would be interesting to evaluate whether increasing CSF1 in the aged CNS can also exert a therapeutic effect on the innate immune activation and glymphatic flow via alterations in the morphology and/or function of meningeal lymphatic vasculature.

Future studies will have to be conducted to continue expanding our understanding about the putative role(s) of adaptive and innate immune changes on the progressive decay of the meningeal lymphatic drainage in aging; determining the exact mechanism(s) as well as the timing of such detrimental immune changes will be crucial to prevent the dampening of CNS cleansing by the glymphatic system and the meningeal lymphatic vasculature.

## Overview on the neuropathological and immune components of AD

### Pathological hallmarks and immune-related risk factors in AD

AD is an elderly-related neurodegenerative disease that causes personality changes and marked cognitive impairments, including severe memory loss. Classically, the pathological diagnosis of AD is based on the abnormally high levels of two misfolded protein aggregates in the brain: extracellular amyloid beta (Aβ) plaques, and intracellular neurofibrillary tau tangles (Fig. 2). Besides these two main pathological hallmarks, the brains of AD patients often present cerebral amyloid angiopathy, vascular damage, accumulation of other types of protein aggregates (e.g., Lewy bodies or TAR DNA-binding protein 43 inclusions), exacerbated glial activation, and marked cell loss (reviewed in greater detail in [100, 101]). This disease can be further divided in two main categories: early-onset AD (EOAD, also called familial AD) and late-onset AD (LOAD, also called sporadic AD). EOAD is quite rare (represents less than 5% of all AD cases) and is attributed to autosomal dominant mutations in one of three genes, namely the amyloid precursor protein (*APP*), presenilin 1 (*PSEN1*), and *PSEN2* [101]. LOAD accounts for the overwhelming remainder of the cases and is quite heterogeneous in nature. Aging is the main risk factor for LOAD, but there are multiple environmental and genetic factors that might increase the odds of developing dementia later in life [88, 101–103].

Impaired neurovascular coupling or functional hyperemia is a prominent feature of the AD brain. This is thought to be in part attributed to the deposition of toxic Aβ oligomers around brain blood capillaries, and consequently to a local increase in the levels of reactive oxygen species, contraction of pericytes, constriction of the capillaries, and ultimately in a reduction of cerebral blood flow (CBF) [104]. Another explanation for reduced functional hyperemia in AD is a faulty activation of neuronal N-methyl-D-aspartate receptors, which then leads to lower production of potent vasodilator nitric oxide. Interestingly, this deficit in N-methyl-D-aspartate receptor-mediated neurovascular coupling in patients and mouse models of brain amyloidosis has been linked to low levels of tissue plasminogen activator [105]. Impaired CBF and decreased spontaneous vasomotion can then further contribute to decreased paravascular clearance of toxic Aβ species from the brain ISF and lead to aggravated cerebral amyloid angiopathy and parenchymal amyloid deposition [106]. Likewise, mice that recapitulate pathological tau accumulation in the brain also display reduced CBF due to impaired signalling between neurons and blood vessels. Decreased neurovascular coupling in a mouse model of tauopathy was linked to a dissociation between neuronal nitric oxide synthase from postsynaptic density 95 and, once again, to reduced production of nitric oxide during glutamatergic synaptic activity, a mechanism that is vital for proper CBF and cognitive function [107]. Recent studies have highlighted alterations in the transcriptomic signatures of vascular cells in six different brain regions from AD patients. Interestingly, vascular cells from the AD brain showed ~ 2,700 differentially expressed genes linked not only to impaired BBB integrity, but also to dysregulated immune responses [108]. Out of these genes, 125 genes were identified as genetic risk variants, and gene ontology analyses pointed to altered IL-17 signalling in fibroblasts, blood endothelial cells, and pericytes as a possible disrupted proinflammatory mechanism in AD patients. Another study relied on single-nuclei RNA-seq of isolated blood vessels from human hippocampi and cortices to show that the expression of 30 AD risk genes, out of the top 45, including phospholipase C gamma 2, phosphatidylinositol binding clathrin assembly protein, and CD2 associated protein, are highly expressed by vascular cells [109]. Altogether, these novel data unveiled an impaired communication between the different cells of the neurovascular unit in AD, namely blood endothelial cells, pericytes, astrocytes, microglia, and neurons that might holistically contribute to reduced neurovascular coupling. Future studies should invest in understanding the mechanistic crosstalk between neurovascular cells, and how its disruption can contribute to the development of AD.

We will dedicate the next few paragraphs to key genetic risk factors for LOAD that have been closely linked to alterations in immune mechanisms. Multiple genome-wide association studies (GWAS) have delineated different genes and gene loci that confer varying degrees of risk for LOAD by affecting primarily innate immune cell responses and functions in the CNS. Amongst the continuously growing list of LOAD common or rare risk variant genes, we can find *APOE*, *TREM2*, *CD33*, ATP binding cassette subfamily A member 7, complement receptor 1, Spi-1 proto-oncogene, membrane-spanning 4A, phospholipase C gamma 2, and others [110–118]. In mammalians, most of these genes, and their encoded proteins, are highly expressed by peripheral and CNS innate immune cells, including microglia (and in some cases by other neural cells), and serve as potent immunomodulators that have profound effects on neuroinflammation and neurodegeneration in AD (reviewed in greater detail in [119–121]). In the next lines we will briefly elaborate on the characteristics and roles of three of the most studied immune-related LOAD risk genes, namely *APOE*, *TREM2*, and *CD33*.

The *APOE* gene encodes for the most abundant brain lipoprotein that in humans is manifested in three major isoforms of ApoE2, Ε3, and Ε4. Of note though, ApoE is also highly produced and secreted by hepatocytes, and plays crucial roles in regulating peripheral lipid homeostasis and preventing atherosclerosis. ApoE3 is the most common isoform (~ 75% of the population express at least one *APOE3* allele) and does not alter the risk for LOAD, whereas ApoE2 is expressed in ~ 5% of the population and confers some degree of neuroprotection against this disease. Expression of *APOE4* represents the greatest known genetic risk factor for LOAD in Caucasian populations, and its effect is dose-dependent, increasing the risk as much as ~ 15-fold in the presence of two *APOE4* alleles. ApoE4 negatively affects different biological processes in the brain such as lipid transport and cholesterol metabolism, glial activation, vascular integrity and blood flow, and Aβ clearance and aggregation, just to name a few (reviewed in greater detail in [122–124]). Interestingly, a recently published study puts forward the idea that ApoE4 can have a negative effect on the brain not only when it is produced locally (by astrocytes and microglia), but also when it is originating from the periphery, namely from the liver. This deleterious effect of liver-derived ApoE4 on the brain was shown to happen via a dysregulation of BBB integrity and function [125]. *APOE4* mice show reduced blood vessel density and length, microvascular damage, loss of pericyte coverage, and signs of DNA fragmentation in both pericytes and blood endothelial cells; all indicative of exacerbated BBB breakdown [126]. Moreover, these mice present reduced neurovascular coupling and resting CBF, features that recapitulate what is also seen in patients carrying at least one *APOE4* allele [127, 128]. Interestingly, these prominent neurovascular deficits were observed in *APOE4* mice even in the absence of Aβ pathology [129].

Heterozygous polymorphisms in the human *TREM2* gene have also been linked to increased risk for LOAD, implicated in altered brain lipid signalling and metabolism, and closely connected to altered CNS innate immunity, due to its elevated expression by resident microglia and BAMs. The most common gene polymorphism is the *TREM2*^*R47H*^, which leads to TREM2 loss-of-function and is linked to a ~3-fold increased risk for LOAD (reviewed in greater detail in [121]). TREM2 is a transmembrane receptor of the Ig superfamily that depends on binding to the adaptor proteins DAP10 or DAP12 for downstream signal transduction [130, 131]. A soluble form of TREM2, whose role is still poorly understood, can also be formed and shed upon enzymatic cleavage by A disintegrin and metalloproteinase domain-containing protein 10 or 17 [132, 133]. TREM2 seems to act as a scavenger receptor, recognizing ligands of assorted natures. Reports have shown that TREM2 has the capacity to bind different lipid and glycolipid species, lipoproteins like ApoE, high or low density lipidated particles, and even aggregated Aβ [134–137].

In 2011, two independent GWAS have identified polymorphisms in the *CD33* gene locus that increase the risk for LOAD [112, 113]. The *CD33* variant rs3865444 is thought to affect *CD33* gene expression, whereas rs12459419, another variant that is regarded as a functional surrogate of rs3865444, has been linked to the splicing of exon 2 of the *CD33* gene [138, 139]. CD33 is a receptor that belongs to the sialic acid-binding Ig-like lectins family and, in humans, is considered an inhibitory transmembrane receptor due to the presence of an immunoreceptor tyrosine-based inhibitory motif in its cytoplasmic domain. This important feature, alongside the high affinity for sulfated sialosides by human CD33, represent two of the major differences between human and murine CD33 biology (reviewed in greater detail in [140]). These are important differences that need to be considered when attempting to interpret experiments involving the manipulation of murine CD33, and highlight the need to develop and use humanized murine lines to better understand the functional role of CD33 in AD. In the human AD brain, there is an increased expression of *CD33* by resident innate immune cells that accompanies the build-up of Aβ plaques, advanced cognitive decline, and overall aggravation of disease severity [141–143]. Interestingly, the same overexpression of *CD33* is also observed in blood monocytes from AD patients [139, 144]. In vitro experiments involving microglial cell lines or blood monocytes suggest that higher levels of CD33 in these distinct myeloid cells lead to similar impairments in activation and phagocytosis of different molecules, including Aβ aggregates [138, 144–146]. Accordingly, initial studies performed using an amyloidosis mouse model deficient for *Cd33* led to the conclusion that decreased signalling via CD33 induces a protective response that is associated with overall lower soluble and insoluble Aβ levels in brain, most likely due to improved microglial activity [147]. But again, the differences in CD33 biology between mice and humans make it hard to judge whether these observations regarding CD33 loss-of-function made in mice are relevant when it comes to the human disease.

In the next sub-section of this review, we will delve deeper into how ApoE, TREM2, and CD33 can modulate CNS innate immunity, with a special emphasis on the regulation of microglial function in AD.

### Innate immune mechanisms that affect AD pathophysiology

Compelling evidence favors the idea that well-tuned innate and adaptive immune mechanisms are vital to nurture a neuroprotective environment, and to curb the emergence of deleterious neuroinflammatory responses that are often linked to neurodegeneration in AD. Parenchymal microglia and BAMs are the innate immune sentinels that constantly survey the brain interstitium for antigens, which in the context of AD can appear as misfolded Aβ and tau fragments, toxic lipid species, cell debris, or intracellular content released upon cell death (reviewed at a greater extent in [32, 120, 121, 148]). In the presence of such triggering stimuli, these brain resident innate immune cells adopt an activated morphology, phagocytose and present processed antigens, and sound the alarm by secreting inflammatory cytokines. These effects might be protective, for instance when microglia eliminate apoptotic cells, clear misfolded Aβ, or release neurotrophic growth factors to promote tissue regeneration; but they can also be harmful, namely when microglia remain chronically activated and contribute to the establishment of an unwanted inflammatory milieu that fosters AD-like brain pathology, impaired synaptic activity, and neuronal loss. A unique state of chronically activated microglia, named disease associated microglia (DAM), has been initially identified using transgenic mouse models of EOAD-like brain amyloidosis (e.g., 5xFAD mice) and state-of-the-art RNA-seq techniques [149, 150]. Interestingly, the gene expression signatures associated with the DAM phenotype, or other well-defined microglial activation status (like the white matter-associated microglia), have now been extensively characterized and found in aged mice, in addition to models of other neurodegenerative disease, like multiple sclerosis, amyotrophic lateral sclerosis, and primary tauopathy [149–152]. Employing scRNA-seq, researchers have also shown that the acquisition of the DAM signature in the murine brain is highly dependent on the expression of murine *Trem2* and *Apoe*, and seems to follow a two-step process [149, 150, 153]. Importantly, apart from the lack of consensus regarding the existence of a true DAM signature in the human AD brain, several comprehensive transcriptomic datasets suggest that human microglia acquire signatures that resemble some features of the DAM phenotype, and that these activation signatures might play relevant roles when it comes to AD pathogenesis and/or progression in humans [154–157]. Data obtained with either *Trem2*^*–/–*^ or humanized *TREM2*^*R47H*^-expressing mice (that also causes TREM2 loss-of-function) seem to suggest that the TREM2-dependent DAM phenotype is protective when it comes to constraining Aβ plaques, preventing the spread of toxic Aβ species, and averting neuronal death and cognitive decline [134, 158–161]. Yet, more work is needed to fully understand the exact role of DAMs in the containment of AD brain pathology and, more importantly, in determining their role in the development of, or resilience to, cognitive decline.

Recent studies highlighted the existence of an intracellular crosstalk between the TREM2 and CD33 signaling pathways, and suggest that, at least in mice, signaling downstream of CD33 induces a deleterious microglial activation status that counteracts the protective effects of TREM2 [159]. Expression of CD33 promotes Aβ accumulation and deposition and is somehow counteracted by the protective signaling via the complex formed by TREM2 and the adaptor protein DAP12 in CNS innate immune cells, including microglia [134, 159–161]. The tyrosine kinase SYK is another important player in the TREM2 signalling pathway. By deleting SYK under a myeloid cell promoter, researchers have shown that microglia do not fully convert into the DAM phenotype, cannot properly phagocytose, and that, altogether, this has a negative impact on Aβ burden and cognition in mouse models of brain amyloidosis [158, 162]. It was noted, however, that *Syk*^*–/–*^ microglia could still proliferate and upregulate *Apoe* due the activation of a SYK-independent pathway that involved DAP10, another adaptor protein that also binds to TREM2 [158]. This type of comprehensive studies are crucial to continue disentangling the beneficial (or detrimental) role(s) of TREM2 and CD33, and respective intracellular signalling pathways, in murine models of amyloidosis, tauopathy, or mixed pathologies [134, 151, 163–165]. Like TREM2, ApoE is also a master regulator of microglial fitness and activity in AD [123, 148]. Different studies have highlighted a close relationship between ApoE and TREM2 when it comes to regulating brain phagocyte function in AD. Both *Apoe*^*–/–*^ and *Trem2*^*–/–*^ mice show impaired microglial clustering around Aβ plaques, which leads to the occurrence of more diffuse and toxic Aβ plaques [166, 167]. Moreover, the decreased Aβ plaque containment by microglia, and increased plaque seeding in the brains of *Trem2*^*–/–*^ mice is closely linked to decreased levels of Aβ plaque-associated ApoE [167], suggesting that both TREM2 and ApoE modulate a protective response by microglia in the context of increased brain Aβ burden.

Replacement of endogenous murine *Apoe* by *APOE4* in humanized knock-in mice results in aggravated Aβ seeding, plaque load and tau pathology in the brain, effects that are to a great extent mediated by the deleterious responses fostered by ApoE4 not only in microglia, but also in astrocytes and blood vessels. Due to its pleotropic deleterious effects, it is with no surprise that ApoE4 has been closely associated with accelerated neuronal loss and cognitive decline in models of AD [129, 149, 150, 168–171]. In a model of tauopathy, widespread ablation of myeloid cells that depend on CSF1R signalling (including microglia, BAMs, and dendritic cells) was shown to almost completely prevent tau-mediated neurodegeneration in mice expressing *APOE4* [172], suggesting that CNS innate immunity might be playing opposite roles when it comes to controlling Aβ pathology or tau-driven neurodegeneration. Interestingly, in *APOE4* mice, microglia drive tau-mediated neurodegeneration independently of TREM2 by acquiring a harmful proinflammatory phenotype characterized by increased lysosomal activity, aerobic glycolysis, and impaired lipid metabolism (with accumulation of intracellular lipid droplets) [164, 170, 172, 173]. It is still unclear whether ApoE4 also affects the function of CNS innate immune cells other than microglia, namely the BAMs and dendritic cells that are found at the brain meninges, and if dural immune cells are somehow implicated in the formation of tau-mediated neuroinflammation and neuronal death.

Microglia have been centre stage when it comes to brain immunity in AD. However, there is an ever-growing body of evidence that points to circulating innate myeloid cells, such as neutrophils and monocytes, and brain resident PVMs as important players in AD. Circulating neutrophils exert a serious impact on meningeal and brain blood flow, wreaking havoc without ever entering the brain parenchyma [174]. In an AD mouse model of brain amyloidosis, neutrophils adhere to the luminal side of small blood capillaries, clogging them and causing blood flow stalling. Depletion of neutrophils reduced the number of stalled capillaries, improved cerebral blood flow, and enhanced the animal’s performance in memory tests [174]. It is still debatable whether peripheral monocytes are recruited into, and engraft the brain in models of AD, particularly in models of brain amyloidosis. There is some consensus, however, regarding the beneficial role of recruited CCR2^+^ monocytes and brain PVMs in clearing vascular Aβ, despite their apparent lack of interaction with parenchymal Aβ plaques [161, 163, 175–178]. CCR2^+^ monocyte-derived macrophages recruited into the brain have also been shown to mediate the precognitive effects of anti-PD-L1 treatment in *Trem2*^*–/–*^ 5xFAD mice [179]. Interestingly, a recent publication generated and integrated different scRNA-seq datasets of brain innate myeloid cells, developed new computational analysis tools, and employed elegant fate-mapping models to confirm the presence of the embryonically yolk sac-derived DAM and to uncover another disease-associated transcriptionally distinct population of monocyte-derived macrophages in the aged and diseased brain [153]. Authors named the later disease inflammatory macrophages and showed that, despite the high TREM2 expression, and contrarily to the neuroprotective DAMs, these macrophages expand in mice lacking TREM2, secrete TNF and might play a deleterious role by promoting a neuroinflammatory environment that further accelerates amyloid pathology and brain damage [153]. Brain PVMs have also been implicated in the early development of cerebrovascular damage provoked by toxic Aβ species (in a CD36 and NADPH oxidase 2 dependent manner) in different mouse models of exacerbated brain amyloidosis, as well as in the deleterious elimination of synapses by microglia [180, 181]. In an APP knock-in mouse model, brain PVMs secrete osteopontin, which is essential for the priming of adjacent microglia into an activated, phagocytic, and complement-secreting phenotype that ultimately leads to aberrant synaptic pruning [181]. Taking into consideration the recent work implicating the skull bone marrow as a source of myeloid cell precursors in the meningeal dura and brain, it will be essential to determine whether this process affects BAMs’ turnover and function in rodent models of AD amyloidosis and tauopathy [42, 47, 182]. We might only be scrapping the surface when it comes to the origin and role(s) of brain innate immune cells in AD.

### Roles of adaptive immune cells in AD

Considering the intricate functional connection between innate and adaptive immune cells, one can assume that perturbations in one arm of the immune system will seriously impact the other, even when it comes to brain immunity in AD. Data obtained using *postmortem* brain samples from AD patients point to an accumulation of extravascular T cells in the brain, more specifically in the hippocampus, parenchymal regions adjacent to the CSF-filled ventricles, and brain border tissues like the leptomeninges [35, 183–185]. Remarkably, CD45RA^+^ effector memory CD8 T cells (also called TEMRA CD8 T cells) are increased in the CSF and blood of patients diagnosed with mild cognitive impairment or AD, showed a higher propensity to secrete IFN-γ, and their numbers were positively correlated with worse cognitive performance, thus indicating its potential value as a biomarker of disease progression and severity [35]. Increased parenchymal T cell numbers have also been observed in AD patients diagnosed with more advanced Braak stage [185]. Moreover, increased T cell receptor clonality in the CSF of AD patients is suggestive of an antigen specific response. Whether there is indeed an expansion of T cells recognizing Epstein-Barr viral antigens, like the data presented in this study seems to suggest, will need to be further validated in independent cohorts of patients [35]. In a mouse model of tauopathy expressing *APOE4*, increased T cell receptor clonality was observed within brain infiltrating activated CD4 and CD8 T cells. Researchers also observed an accumulation of these activated T cells in the hippocampus, in close proximity to microglia that expressed high levels of MHC-II [185]. This unexpected infiltration of antigen-specific T cells was quite evident in this primary tauopathy model expressing ApoE4 but absent in their age-matched counterparts with exacerbated brain amyloidosis, an observation that might be explained by the overt neurodegeneration that is observed in the first model but only minimal in the later [185]. Notably, widespread depletion of myeloid cells in the brain and its border tissues, using a CSF1R antagonist, lessened the T cell infiltration, suggesting that antigen presentation by APCs, by MHC-II^high^ microglia and/or hypothetically by dural DCs, is necessary for the phenomena of activated T cell extravasation and retention in this model of AD-like neurodegeneration [185]. Similarly to what was observed upon treatment with a CSF1R antagonist, prolonged depletion of T cells (via injections of anti-CD4 and anti-CD8 depleting antibodies) reduced the levels of hyperphosphorylated tau and reduced neuronal loss in the *APOE4*-expressing tauopathy mouse model [164, 172, 185]. The authors put forward the idea that increased levels of CCL3, CCL4, and CXCL10 in the brains of mice undergoing neurodegeneration might be at the genesis of peripheral T cell recruitment, but more experiments need to be performed to determine the molecules behind the T cell recruitment, the possible contribution of neurovascular injury to T cell extravasation, the molecular cues driving the expansion of the specific T cell receptor clones, and the exact mechanisms through which activated microglia and T cells promote neuronal death. Interestingly, this study also shows an expansion of CD8 and CD4 T cells, as well as Tregs, in the meninges of mice with overt neurodegeneration induced by tau and ApoE4 [185]. This is somewhat in accordance with the increases in dural effector T cells and Tregs that is observed in the meningeal dura of very old mice, which also present reduced meningeal lymphatic drainage, and of adult CCR7^–/–^ 5xFAD mice, which present impaired immune cell lymphatic egress to the cervical LNs [18, 37]. Future studies should be designed to test whether the meningeal lymphatic system has any role to play in the formation of the aberrant T cell responses observed in this model of primary tauopathy with neurodegeneration.

Modulating T cell activation via immune checkpoint blockade has been seen as a promising therapeutic strategy to reset CNS immunity and prevent neuronal damage in AD. In particular, administration of antibodies against program cell death protein 1 (PD-1), which is highly expressed in activated/exhausted T cells, or against its ligand PD-L1, has generated very interesting yet controversial data [186–188]. Injections of anti-PD-1 or anti-PD-L1 have been linked to the expansion of IFN-γ-producing T cells in the blood and at brain border tissues, enhanced recruitment of peripheral monocytes into the brain, and alleviation of either brain Aβ plaque load, or tau pathology in different AD mouse models [186, 187]. Despite the lack of reproducibility regarding the beneficial effects of anti-PD-1 immunotherapy in reducing Aβ plaques in models of brain amyloidosis, injections of anti-PD-1 antibodies were also able to significantly reduce neurodegeneration in mice expressing tau and *APOE4* [185, 188]. Interestingly, the protective outcome in this model of tauopathy with ApoE4 expression was associated with a higher frequency of Tregs in the brain without any changes in effector T cells, which suggests that the protective mechanisms of anti-PD-1 might be disease context-dependent [185]. More work is needed to fully grasp the protective mechanisms induced by such therapeutic approaches involving immune check point inhibitors.

An increasing number of publications strongly supports the role of B cell-mediated immunity in AD. A small cohort of patients with higher cerebral Aβ levels show increased numbers of B cells and higher IgG levels in the blood. Interestingly, assessment of the B cell receptor repertoire in these AD patients revealed a lower clonal diversification, which might be indicative of increased B cell clonality [189]. Future studies should use larger cohorts of AD patients to evaluate the presence of ABCs [43], and whether these clonally expanded B cells that arise with aging are in fact increased in the blood and/or brain. Notably, circulating B cells from AD patients secrete IgG that can impair microglial activation in vitro, leading to decreased capacity to phagocytose Aβ [189]. Likewise, a study involving 5xFAD mice, revealed significantly increased B cells in the cervical LNs and higher levels of IgG associated with microglia and Aβ plaques in the brain. Either the genetic depletion of mature B cells or the reduction of B cells by a regimen of anti-CD20 and anti-CD45R/B220 antibodies ameliorated Aβ deposition in the brain, restored the levels of transforming growth factor beta (TGF-β), and improved the exploratory and spatial memory function of AD transgenic mice [190]. However, contrarily to what these previous studies propose, brain Aβ accumulation and deposition was greatly accelerated in immune compromised mice that lacked mature B and T cells, along with NK cells [191]. This was shown to happen, at least in part, due to the loss of B cell-derived IgG, which was proven to be instrumental in promoting a protective microglial phagocytic phenotype that was necessary for proper Aβ clearance. On the same line, a recent publication has shown that depletion of mature B cells, initially shown to be increased in the brain and meninges of 5xFAD mice, led to aggravated brain Aβ pathology and accelerated cognitive decline. This deleterious effect was linked to decreased levels of IL-35, a cytokine that is supposedly secreted by said protective B cells and modulates neuronal Aβ production [192]. Taken together, these data suggest that B cells and their secreted Igs have the capacity to influence microglial function and EOAD-like brain amyloidosis; but there is still some disagreement regarding the nature of their role, and whether B cell responses need to be arrested or fostered to improve brain function in the context of AD.

## Sorting out the roles of meningeal lymphatic drainage and immunity in AD

For a while now, and as we discussed in prior sections, researchers have been dedicating substantial effort and resources in understanding the role of the immune system in AD. In the last decade or so, there has been a growing interest about the changes taking place at the meninges, which represent a yet unexplored neuroimmune hub that facilitates the interaction between brain antigens and the peripheral immune and lymphatic vascular systems. Most of the recent advances on this topic were possible through experiments involving transgenic mouse models that recapitulate the overt brain amyloidosis that is seen in EOAD and is thought to manifest decades prior to the onset of clinical symptoms in LOAD [101, 193]. The impact of aging on meningeal lymphatic morphology and immunity is accelerated in models that present exacerbated brain Aβ production. Middle-aged 13–14-month-old 5xFAD mice showed reduced meningeal lymphatic vascular coverage that coincided with higher numbers of dural T and B cells and, interestingly, with evident Aβ vascular deposition in this outmost meningeal layer [22] (Fig. 2). By inducing this aging-like meningeal lymphatic vessel disruption in young-adult AD transgenic mice for a prolonged period, researchers were able to show that decreased meningeal lymphatic drainage led to exacerbated deposition of Aβ in the meninges and the brain [18, 22]. This outcome is thought to result from impaired Aβ glymphatic efflux due to reduced meningeal lymphatic function, lack of appropriate drainage of Aβ from the CSF into the deep cervical LNs, and the establishment of a deleterious meningeal and brain inflammatory responses [18, 21, 22, 37, 194, 195]. Interestingly, adult 5xFAD mice with ablated meningeal lymphatics showed exacerbated dural macrophage activation, less microglia with homeostatic gene expression, and an intensified DAM gene signature in the brain parenchyma, when compared to age-matched transgenic mice with intact meningeal lymphatics [18, 22]. In fact, this exacerbated DAM signature was also observed in 5xFAD mice deficient for CCR7, a receptor that is expressed by meningeal immune cells (but not expressed by microglia) and caused a major impact on dural adaptive immunity, particularly in T cell activation [37]. Interestingly, both the ablation of meningeal lymphatic vessels, which prevents the drainage of brain-derived molecules into the cervical LNs, as well as the prevention of meningeal immune cell lymphatic egress by deletion of CCR7, culminated in dampened glymphatic function and worsened cognition in adult male 5xFAD mice [22, 37]. As we mentioned earlier, it was recently shown that altered activation of brain BAMs is directly linked to changes in vascular fitness and glymphatic function [99]. Future studies should be designed to understand whether the decrease in perivascular glymphatic influx/efflux observed after impairing meningeal lymphatic drainage is mediated by the exacerbated activation of BAMs and/or microglia. Published data also strongly suggests that lengthy alterations in meningeal lymphatics in AD transgenic mice lead to abnormal T and B cell responses that ultimately modulate innate myeloid cell activation in the brain [18, 22, 37, 191]. However, more work is needed to determine the exact molecular player(s) mediating such communication between meningeal immune and brain cells (e.g., IL-10, TGF-β, IFN-α, IFN-β, or IFN-γ), and whether specific meningeal immune cells and/or inflammatory molecules can be targeted to promote a protective neuroinflammatory state that favors Aβ clearance and prevents cognitive decline.

Alongside Aβ, the meningeal lymphatic system also aids in the elimination of toxic tau species from the brain. Mice that lacked a functional dural lymphatic vascular system showed a delayed clearance of monomeric tau injected into the brain parenchyma [196]. Likewise, a recent publication further corroborates the outflow of tau species from the brain into the cervical LNs mediated by the glymphatic system and the meningeal lymphatic vasculature [197]. Authors go on to show that deficiency in aquaporin 4 (AQP4), a channel protein expressed at the astrocytic endfeet that is essential for appropriate glymphatic function [24, 25, 198, 199], aggravates tau hyperphosphorylation and neurodegeneration in a model of tauopathy. Of note, and surprisingly, the drainage of tau from the CSF into the deep cervical LNs is severely compromised in mice that lack AQP4 [197]. This is somewhat puzzling, because the process of CSF content drainage into the cervical LNs is solely mediated by lymphatic vasculature, and at a great extent by the meningeal lymphatic vessels [1, 10, 30]. Taking into consideration that the authors used constitutive *Aqp4*^*–/–*^ mice, it will be important to investigate whether there are defects in meningeal lymphatic morphology and function in this mouse model that might explain such outcome. It will also be essential to understand whether the clearance of extracellular Aβ or tau in *Aqp4*^*–/–*^ mice, which present constitutive defects in glymphatic function, can be therapeutically enhanced by increasing the levels of pro-lymphangiogenic VEGF-C [18, 22]. On the other hand, little is known about the integrity of the meningeal lymphatic vasculature in mouse models of primary tauopathy. In fact, the heightened meningeal T cell activation and the deleterious T cell infiltration into the brain of *APOE4* mice with overt tau pathology further stresses the need for experiments to evaluate meningeal lymphatic function in mouse models of tauopathy [185]. These new data feeds into the hypothesis that altered meningeal lymphatic drainage, and altered immune responses at the draining cervical LNs, can contribute to the formation of these harmful encephalitogenic T cell responses that promote neuronal loss in the context of tauopathy. On the same line, the reported effects of *APOE* genotype on the formation of the harmful T cell responses underlines the need to better understand if and how certain AD genetic risk factors, being that *APOE*, *TREM2*, *CD33*, or other gene variants, affect the CNS-draining lymphatic vasculature or the immune responses at the cervical LNs.

Studies involving two monoclonal antibodies against toxic Aβ species—aducanumab and lecanemab—have spawned considerable excitement (alongside some controversy) and are currently at the forefront when it comes to therapies for AD patients. However, there is still limited evidence supporting the efficacy of anti-Aβ passive immunotherapy in thwarting cognitive decline, concerns about the development of side effects (e.g., brain microbleeds and amyloid-related imaging abnormalities) that might compromise the final therapeutic outcome, and apprehension about the high cost of such treatments [200–202]. Administration of the murine version of aducanumab to 5xFAD transgenic mice at young ages resulted in lower Aβ plaque burden but did not influence the animals’ performance in cognitive tasks [22]. On the other hand, submitting 5xFAD mice with poor meningeal lymphatic function to a similar murine aducanumab regimen led to significantly higher Aβ plaque burden at the end of the experiment. This worse outcome was not due to a lower efficacy of aducanumab, but instead due to an accelerated deposition of Aβ in the brain in mice with impaired brain lymphatic drainage. Ablation of meningeal lymphatics negatively impacted vascular and microglial activation, increased the occurrence of brain microbleeds, and weakened the cognitive abilities of 5xFAD mice, even after the treatment with aducanumab [22]. However, the exact cellular mechanisms underlying the accelerated Aβ accumulation in mice with deficient meningeal lymphatic drainage remain elusive and need to be further investigated. Interestingly, combining a meningeal lymphatic vessel-targeted therapy, consisting of VEGF-C delivery via a viral vector, with injections of the murine versions of either aducanumab or lecanemab, improved the clearance of Aβ plaques and tempered brain innate immunity in two distinct mouse models of brain amyloidosis [22]. These data support the potential of developing approaches that combine the therapeutic fine tuning of meningeal lymphatic drainage with treatments that are directed towards Aβ clearance or microglial activation in AD.

As we have previously mentioned, aside from the most studied genetic risk factors and neuropathological agents, namely Aβ and tau, certain environmental factors have also been strongly linked to AD development and accelerated progression [101]. Different epidemiological studies have demonstrated an increased risk for long-term dementias, including AD, following traumatic brain injury (TBI) (reviewed in [102]). Recently published data obtained using preclinical mouse models disclosed previously unappreciated associations between the outcomes of TBI and brain bleeding and meningeal lymphatic vascular function [203, 204]. Mice that received a single hit to the right inferior temporal lobe (consider by some as a mild model of TBI), exhibited impaired meningeal lymphatic drainage of CSF molecules into the cervical LNs, as early as 2 h post injury, that was accompanied by a pathological expansion of meningeal lymphatic vessels. This defect in brain lymphatic drainage took roughly 1 month to be restored back to baseline levels, highlighting to the long-lasting effects of a single mild traumatic hit to the brain on meningeal lymphatic drainage [203]. Administration of the pro-lymphangiogenic VEGF-C to aged mice post-TBI led to a better outcome in terms of brain gliosis, suggesting that early promotion of meningeal lymphatic drainage upon TBI might have therapeutic benefits [203]. On the same line, impaired meningeal lymphatic drainage has been linked to poor cortical perfusion, worsened clearance of erythrocytes, and exacerbated brain edema and neuroinflammation in a rodent model of subarachnoid hemorrhage. This delayed recovery from brain hemorrhage due to poor lymphatic drainage led to aggravated cognitive deficits, emphasizing, once again, the importance of promoting proper meningeal lymphatic drainage early on upon brain injury [204, 205].

Higher levels of infectious agents, like viruses (e.g., herpes simplex virus type 1), have been detected in the brains of AD patients and linked to the development of brain pathology and cognitive impairment [206–208]. Likewise, persistent infections by bacteria, namely the strains causing periodontitis, have also been linked to AD cases and shown to affect AD-like pathology in mice [209, 210]. However, the direct link between infections and the development of AD remain a topic of research and debate [103]. Interestingly, mice infected with neurotropic viruses, including the Japanese encephalitis virus, showed an abnormal expansion of meningeal lymphatic vessels, which was evident at 3 days upon infection, that was accompanied by decreased CSF drainage into the cervical LNs [211]. Of note, a similar acute phenotype of aberrant meningeal lymphangiogenesis has been observed in mice shortly after TBI [203]; future studies should focus on understanding whether similar (or distinct) molecular mechanisms govern the expansion of meningeal lymphatics in these two different models of brain neuroinflammation and pathology. Mice infected with neurotropic viruses presented a concomitant increase in VEGF-C and proinflammatory cytokines in the brain, such as IL-6 and TNF. These data suggest that alterations in inflammatory mediators can be underlying the observed pathological expansion of meningeal lymphatics and lead to a reduced drainage of viral particles to the cervical LNs, a process that was proven essential to combat these infectious neurotropic viruses [211]. The administration of VEGF-C prior to viral infection prevented the deleterious effects on the meningeal lymphatic vasculature, reduced the viral load in the brain and improved survival [211]. Future work should focus on the effects of CNS chronic infection on meningeal lymphatic functions. Whether long-term changes in brain lymphatic drainage due to infection, either acute or chronic, can affect the risk for AD remains an interesting open question that should be addressed in years to come.

## Concluding remarks

The presence of a functional meningeal lymphatic system that mediates the drainage of CNS antigens and meningeal immune cells into the cervical LNs only bolstered the concept that altered neuroimmune responses at the brain borders and parenchyma play an active role in the progression of brain pathology and neural dysfunction in AD. As we pointed throughout this review manuscript, significant advances have been made since the (re)discovery of the meningeal lymphatic system. There is now considerable evidence, stemming from independent groups, showing the negative consequences of meningeal lymphatic vessel impairment in AD models, and the remarkable benefits of restoring meningeal lymphatic drainage when this specialized vascular system is performing below physiological levels [18, 22, 194, 212–215]. However, more work needs to be performed to fully unveil the benefits of enhancing meningeal lymphatic drainage in models that recapitulate features of AD brain pathology other than amyloidosis or tau hyperphosphorylation and aggregation. Based on published data showing profound alterations in peripheral tissue lymphatics in *Apoe*^*–/–*^ mice, which develop hypercholesterolemia and atherosclerosis with aging, it would be very interesting to understand the effects of *APOE4* on the meningeal lymphatic system [216]. Our lack of knowledge about the molecular mechanisms governing meningeal lymphatic vessel fitness in aging and AD must be filled in years to come, so that we can develop even better personalized therapies to modify meningeal lymphatic function (e.g., promote lymphatic vessel growth), either in the context of amyloidosis, tauopathy, mixed pathologies, or expression of known LOAD genetic risk factors.

The first steps have been taken towards developing non-invasive MRI techniques to assess the meningeal lymphatic coverage and drainage capacity in humans [10, 12, 95, 217]. It will be critical to continue optimizing such techniques, implement them routinely into the clinical setting, and use them to comprehensively determine if meningeal lymphatic vascular dysfunction is in fact a pathological phenomenon observed in AD patients, how early it can be detected, and whether it is linked to changes in other AD pathological hallmarks and/or biomarkers. Defective meningeal lymphatic drainage, alongside abnormal meningeal immunity and inflammation, might represent previously unappreciated pathological events that, if detected in time, might be easily therapeutically targeted to preserve brain function and prevent cognitive decline in AD.

In sum, a generalized effort should be put in place to 1) determine the environmental and genetic factors that affect meningeal lymphatic vascular function, 2) understand whether and how meningeal lymphatics and immunity are implicated in AD pathophysiology, and 3) identify specific meningeal cellular and molecular players that can be contemplated as easily accessible therapeutic targets. It is urgent to develop efficacious single or combinatorial treatments to resolve ongoing deleterious immune reponses, neuroinflammation and prevent neurodegeneration in this devastating, and ever more prevalent, type of dementia.

## Data Availability

Not applicable.

## Abbreviations

Aβ: Amyloid beta
ABCs: Age-associated B cells
AD: Alzheimer’s disease
APCs: Antigen-presenting cells
ApoE: Apolipoprotein E
APP: Amyloid precursor protein
AQP4: Aquaporin 4
BAMs: Border-associated macrophages
BBB: Blood-brain barrier
CBF: Cerebral blood flow
CCL: C–C motif chemokine ligand
CD: Cluster of differentiation
CNS: Central nervous system
CCR: C–C motif chemokine receptor
CSF: Cerebrospinal fluid
CSF1R: Colony stimulating factor 1 receptor
CXCL: C–X–C chemokine ligand
CXCR: C–X–C chemokine receptor
DAMs: Disease-associated microglia
DCs: Dendritic cells
EOAD: Early onset Alzheimer’s disease
GWAS: Genome wide association studies
IFN-α/β/γ: Interferon alpha/beta/gamma
Ig: Immunoglobulin
IL: Interleukin
FTY720: Sphingosine-1-phosphate receptor inhibitor
LECs: Lymphatic endothelial cells
LNs: Lymph nodes
LOAD: Late onset Alzheimer’s disease
LYVE-1: Lymphatic vessel endothelial hyaluronan receptor-1
MAIT: Mucosal-associated invariant T
MHC-I or II: Major histocompatibility complex class I or II
MOG: Myelin oligodendrocyte glycoprotein
MRI: Magnetic resonance imaging
NK: Natural killer
PD-1: Program cell death protein 1
PROX1: Prospero homeobox 1
PSEN1 or 2: Presenilin 1 or 2
PVMs: Perivascular macrophages
scRNA-seq: Single-cell RNA sequencing
SYK: Spleen associated tyrosine kinase
TBI: Traumatic brain injury
TCRγδ: T cell receptor gamma/delta
TGF-β: Transforming growth factor beta
TNF: Tumor necrosis factor
TREM2: Triggering receptor expressed on myeloid cells 2
VEGF-C: Vascular endothelial growth factor C
VEGFR3: Vascular endothelial growth factor receptor 3
